# Arsenic-Associated Changes to the Epigenome: What Are the Functional Consequences?

**DOI:** 10.1007/s40572-013-0002-8

**Published:** 2014-01-19

**Authors:** Kathryn A. Bailey, Rebecca C. Fry

**Affiliations:** Department of Environmental Sciences and Engineering, UNC Gillings School of Global Public Health, University of North Carolina at Chapel Hill, Campus Box 7431, Chapel Hill, NC 27599 USA

**Keywords:** Arsenic, DNA methylation, Histone post-translational modifications, MicroRNAs, Epigenetic reprogramming, Prenatal exposure, Epigenome

## Abstract

Inorganic arsenic (iAs) poses a major threat to worldwide human health, and yet the molecular mechanisms underlying the toxic effects associated with iAs exposure are not well understood. There is increasing experimental evidence indicating that epigenetic modifications may play a major role in the development of diseases associated with exposure to environmental toxicants. Research in the field has firmly established that iAs exposure is associated with epigenetic alterations including changes in DNA methylation, miRNA abundance, and post-translational histone modifications. Here, we summarize recent studies that have expanded the current knowledge of these relationships. These studies have pinpointed specific regions of the genome and genes that are targets of arsenical-induced epigenetic changes, including those associated with *in utero* iAs exposure. The recent literature indicates that iAs biotransformation likely plays an important role in the relationship between iAs exposure and the epigenome, in addition to the sex and genetic background of individuals. The research also shows that relatively low to moderate exposure to iAs is associated with epigenetic effects. However, while it is well established that arsenicals can alter components of the epigenome, in many cases, the biological significance of these alterations remains unknown. The manner by which these and future studies may help inform the role of epigenetic modifications in the development of iAs-associated disease is evaluated and the need for functional validation emphasized.

## Introduction

Exposure to arsenic is a major global threat to human health. The most common route of exposure worldwide is through the consumption of drinking water contaminated with naturally-occurring, geologic sources of inorganic arsenic (iAs) [1]. Although the World Health Organization (WHO) has recommended that levels of iAs in drinking water should not exceed 10 ppb, it is estimated that over 100 million people worldwide are exposed to iAs levels in drinking water that are considered detrimental to human health [2, 3]. Numerous chronic conditions, such as cardiovascular and peripheral vascular disease, diabetes mellitus, neurological effects, and cancers of the skin, lung, liver, urinary bladder and prostate, may develop after long-term iAs exposure [4]. Much remains to be learned about the factors that influence susceptibility of chronically-exposed individuals to these varied adverse health effects, but they likely include individual genetic background, nutritional status, toxicant co-exposure, the dose and duration of iAs exposure, and capacity of individuals to biotransform iAs to reactive metabolites [5, 6].

The molecular mechanisms underlying the diverse health effects associated with iAs exposure are not well understood and are likely complex. Numerous toxic effects have been attributed to exposure to iAs and its reactive metabolites. For instance, the generation of reactive oxygen species (ROS) is believed to play a major role in mediating the toxic effects of various arsenicals by causing biomolecular damage and the activation of cellular signaling pathways that promote disease development [7]. However, as no single mechanism has been identified to date as a key event in the development of any iAs-associated disease, it is plausible that several mechanisms are involved, possibly acting sequentially, simultaneously, and/or synergistically.

In recent years, there has been increased recognition of the importance of the epigenome in maintaining cellular homeostasis, and therefore it has been hypothesized that environmentally-induced epigenetic perturbations may play an important role in disease development. Detailed descriptions of the various components of the epigenome are outside the scope and purpose of this work but are the subjects of reviews elsewhere [8, 9]. Briefly, the epigenome refers to potentially heritable biological information contained outside the DNA sequence that functions as regulators of gene function [8–10]. The most extensively-studied components of the epigenome include DNA methylation and covalent histone post-translational modifications (PTMs), which regulate gene expression at the transcriptional level. In recent years, microRNAs (miRNAs) have been increasingly recognized as another component of the epigenome. As they regulate mRNA levels primarily at the post-transcriptional level and as they act “above the genome” to control gene expression, they are included in this review. Through their roles in regulating gene expression, each of these components therefore plays a critical role in maintaining cellular homeostasis by interacting in coordinated responses to regulate organismal development, maintain genetic stability, and mediate responses to environmental stressors [11–13].

The recognition that epigenetic changes may mediate the toxic effects of iAs stems in part from the observation that it is a potent carcinogen but not a point mutagen. The long-term health consequences of prenatal iAs exposure also support that iAs may exert adverse effects via epigenetic mechanisms. In both humans and animal models, exposure to iAs *in utero* is associated with increased risk for chronic disease development later in life [14–16]. In animal models, experimental evidence suggested this altered disease susceptibility was not associated with changes in DNA sequence, but instead, persistent changes in the expression and epigenetic landscape of critical genes, including those implicated in chronic disease development [17, 18]. Observations such as these are in line with the hypothesis that environmental influences *in utero* play a major role in the fetal origins of adult disease by causing permanent changes, or “reprogramming,” of the epigenome in the fetal environment that persist throughout life [13, 19]. These epigenetic changes are hypothesized to cause altered disease risk via reprogramming the expression patterns of key genes such as silencing tumor suppressors. Importantly, these altered epigenetic marks can be metastable, and thus the effects of altered gene expression and disease risk have the potential to be transgenerational [20, 21]. Therefore, the association between prenatal iAs exposure and the development of a wide variety of chronic diseases in adulthood is consistent with iAs acting as a prenatal toxicant with widespread epigenetic effects. Various arsenic species have been shown to have effects on the epigenetic landscape outside the fetal environment as well. As recently reviewed, some of these epigenetic effects have been implicated as critical factors in the development of diseases associated with chronic iAs exposure [22].

As the effects of arsenicals on the epigenome have been the subjects of several reviews in recent years [22–24], the purpose of the present review is to provide a critical evaluation of the major trends in the more current literature (2011–2013) in which the relationship between arsenic exposure and the epigenome has been examined. Previous reviews have emphasized the following general findings associated with arsenical exposure: (1) both global and gene-specific changes in DNA methylation levels have been observed, (2) histones are targeted for post-translational modifications, and (3) miRNA levels are perturbed [22–24]. In the present review, we expand upon this information and synthesize and evaluate the current state of knowledge of arsenic-associated epigenetic modifications, particularly in the context of their biological significance in disease development. While epigenetic perturbations associated with iAs exposure were first reported in the 1980s [25, 26], two papers published by the Mass and Waalkes laboratories in 1997 marked the beginning of intensive investigations into the effects of arsenicals on the epigenome [27, 28]. As discussed in more detail below, while knowledge of arsenical-induced epigenetic effects has advanced greatly over the past 16 years, we are very much at the beginning of understanding the functional consequences of these modifications, including the potential for specific modifications to influence disease susceptibility as well as the potential heritability of these effects.

## Arsenic and DNA methylation

In mammals, DNA is methylated almost exclusively at the cytosine residue of CpG-dinucleotides in which a single methyl group is enzymatically transferred from S-adenosyl methionine (SAM) to generate 5’-CpG-3’ [29]. CpGs are not distributed evenly throughout the mammalian genome, but instead are clustered into two major regions: (1) ~15 % of CpGs are found in regions of euchromatin known as CpG islands and CpG island shores, and (2) ~85 % of CpGs are found in repetitive interspersed regions of DNA (such as transposable elements) in heterochromatin [30–32]. The best-understood functional consequence of CpG methylation is gene silencing at the transcriptional level. In general, a negative relationship between promoter CpG island/CpG island shore methylation and gene expression is observed [33], and CpGs are heavily methylated in transcriptionally-silenced regions of heterochromatin [32]. However, it should be noted the relationship between DNA methylation and gene expression is likely quite complex, as there are numerous instances in which this relationship has been positive or no relationship has been observed [33, 34].

To date, DNA methylation is the most extensively studied epigenetic alteration in association with arsenic exposure, which has included *in vitro* studies, *in vivo* studies, and studies in human populations (as reviewed in [22–24]). Taken together, studies have revealed that DNA methylation patterns can be altered by iAs and/or iAs metabolites at either the global or gene-specific level. DNA methylation changes have been detected in several genes implicated in iAs-associated disease development, such as promoter hypermethylation of tumor suppressors such as cyclin-dependent kinase inhibitor 2A (*CDKN2A*/ *p16*) or tumor protein 53 (*TP53*) (as reviewed in [22, 24]). In addition, global DNA hypomethylation, which has been implicated as a contributing or predisposing factor of carcinogenesis [35], has been observed upon exposure to arsenic species, including cell lines malignantly transformed by iAs *in vitro* [22, 36, 37]. However, in many cases, the biological consequences of these changes (e.g., corresponding changes in mRNA or protein levels) or significance of relationship (e.g., causal relationship between iAs exposure, DNA methylation alterations, and malignant transformation) have not been established. In addition, specific alterations are not consistently observed across various experimental platforms or studies, which may in part reflect the wide range of arsenic doses and exposure times that have been utilized.

Aside from the examination of the promoter DNA methylation status of genes implicated in iAs-associated diseases, a major research focus has been the investigation of prenatal iAs exposure on DNA methylation patterns. There is considerable experimental evidence suggesting that iAs may act as a prenatal toxicant by altering the DNA methylome. This is supported by the observation that similar relationships between changes in DNA methylation patterns and altered disease risk has been observed with exposure to other prenatal toxicants or perturbed prenatal nutritional status [38–42].

**Fig. 1:**
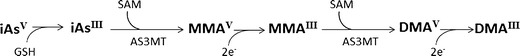
Arsenic biotransformation in humans. Worldwide, the most prevalent source of arsenic exposure in humans is drinking water contaminated with inorganic arsenic (iAs), which exists in two oxidation states, namely pentavalent arsenate (iAs^V^) and trivalent arsenite (iAs^III^). IAs^V^ is reduced to iAs^III^ mainly in the blood or liver by one of several mammalian enzymes that utilize glutathione (GSSH) or dithiothreitol (DTT) as reductants. The remaining steps of iAs biotransformation, which involve alternating oxidative methylation and reduction steps, occur mainly in the liver. In this process, iAs^III^ undergoes an oxidative methylation step catalyzed by arsenic (+3 oxidation state) methyltransferase (AS3MT) to produce monomethylarsonic acid (MMA^V^). MMA^V^ is subsequently reduced to monomethylarsonous acid (MMA^III^), which is the substrate for another round of oxidative methylation and reduction in which dimethylarsinic acid (DMA^V^) and dimethylarsinous acid (DMA^III^), respectively, are produced. AS3MT has been shown to catalyze both the oxidative methylation and successive reduction reactions using s-adenosyl methionine (SAM) as a methyl donor and various molecules such as nicotinamide adenine dinucleotide phosphate (NADPH), thioredoxin, DTT, or GSH as reductants (2e^-^) [43, 45]. In general, total urinary arsenic in iAs-exposed individuals is composed of 10–20 % total (i.e. trivalent + pentavalent) iAs, 10–20 % total MMAs, and 60–80 % total DMAs [44]. (Modified from: Bailey KA and Fry RC, Arsenic-induced Changes to the Epigenome, in Toxicology and Epigenetics, S.C. Sahu, Editor. 2012, John Wiley & Sons, Ltd.: Chichester, UK) [22]

Another major area of interest has been the investigation of the relationship between iAs biotransformation and the DNA methylome. IAs biotransformation involves a series of alternating reduction and oxidative methylation steps that yields six major arsenicals found in the urine of humans and most mammals, namely trivalent and pentavalent forms of iAs and its monomethylated and dimethylated metabolites (MMAs and DMAs, respectively) [43–45] (Fig. 1). These metabolites vary in their toxicities, and as previously mentioned, the efficiency of iAs biotransformation is believed to be an important factor in the development of iAs-associated diseases, as several iAs metabolites are biologically reactive [6]. As the same methyl donor source, namely SAM, is required for both DNA methylation and iAs biotransformation, it has been hypothesized that iAs biotransformation may influence disease risk by altering DNA methylation levels and therefore influencing gene expression patterns [27, 28].

In recent years, examinations of the relationship between alterations in the DNA methylome and prenatal iAs exposure and/or iAs biotransformation have continued as major research thrusts (Table 1). As in earlier studies, many of these investigations have also examined the DNA methylation profiles of genes implicated in the development of iAs-associated diseases. As discussed below, most of these studies have not focused on mechanisms by which iAs may impact the landscape of DNA methylation, but instead have examined the DNA methylation profiles within the white blood cells of iAs-exposed populations, including cord blood of prenatally exposed newborns and peripheral blood isolated from chronically exposed adults.

**Table 1:** Recent studies examining the relationship between DNA methylation and arsenic exposure

Type of study (*in vitro*, *in vivo*, human subjects)	Arsenical exposure	Cell type analyzed	Type of DNA methylation analysis and method used	Major findings	Reference
Women (n = 16) from Zimapán, Mexico	7.46-90.75 μg arsenic/g creatinine in urine	PBLs	Genome-wide analysis of >14,000 promoter regions using the methylated CpG island recovery (MIRA)-microarray assay.	Identified 183 genes with differentially methylated promoters in individuals with arsenic-associated skin lesions vs. those with unlesioned skin. Several of these genes had known relationships with various iAs-associated diseases.	Smeester et al., 2011 [46•]
Women from Argentinian Andes (n = 202)	U-tAs: median=188 μg/L; 5-95 % = 20.8-545 μg/L	PBLs	Analyzed methylation status of regions of *p16* and *MLH1* genes and repetitive LINE-1 elements using bisulfite conversion of DNA followed by PCR amplification and pyrosequencing.	U-tAs positively associated with *p16* and *MLH1* methylation; %iAs in urine positively associated with *p16* methylation; %DMAs in urine negatively associated with *p16* methylation. No associations with LINE-1 methylation.	Hossain et al., 2012 [50•]
Newborns from Thailand (n = 71); *in vitro* exposure to arsenite (lymphoblasts)	Arsenic in cord blood = 0.51-10.37 μg/g; toenail = ND-8.23; hair = ND-0.38 μg/g; *in vitro* short term: 0, 10, 20, 50 μM NaAsO_2_ for 0, 2, 4, 8 h; *in vitro* long term: 0, 0.5, 1 μM NaAsO_2_ for 0, 2, 4, 6, 8 weeks	Cord blood lymphocytes; Lymphoblast cell line RPMI1788	Global DNA methylation was calculated by determination of total 5-methyldeoxycytidine content via HPLC-MS/MS and the DNA methylation status of LINE-1 using combined bisulfite restriction analysis (COBRA). DNA methylation status of *p53* determined by DNA methylation-specific restriction enzyme nuclease digestion.	Positive association between *p53* promoter methylation and toenail arsenic in newborn cord blood. Short-term *in vitro* studies revealed *p53* promoter hypermethylation and global DNA hypomethylation. Long-term *in vitro* studies revealed transient *p53* hypermethylation and slight global DNA hypomethylation.	Intarasunanont et al., 2012 [52]
Mother-newborn pairs from Bangladesh (n = 114)	Maternal U-tAs = 0.04-21.87 μg/g creatinine	Peripheral blood leukocytes (mothers); cord blood leukocytes (newborns)	Analyzed methylation status of regions of *p16* and *p53* genes and LINE-1 and Alu 1 repetitive elements using bisulfite conversion of DNA followed by PCR amplification and pyrosequencing.	In both mothers and newborns, positive association between maternal U-tAs and LINE-1 methylation and some CpG sites within *p16* promoter. Results suggest moderate levels of iAs exposure can impact DNA methylome.	Kile et al., 2012 [53]
Newborns from New Hampshire (n = 134)	Maternal U-tAs = 1.8-6.6 μg/L	Cord blood	Genome-wide DNA methylation analysis of >485,000 CpG sites at single-nucleotide resolution using bisulfite conversion of DNA followed by hybridization to the Illumina Infinium HumanMethylation450 BeadChip.	Associations between maternal U-tAs and methylation status of several CpG sites identified, several of which had linear relationships. Results suggest low levels of iAs exposure can impact DNA methylome.	Koestler et al., 2013 [54]
Mother-newborn pairs from Bangladesh (n = 101)	Maternal U-tAs mean = 271.7 μg/g creatinine	Cord blood	Analysis of global DNA methylation analysis using several methods: ^3^H-methyl incorporation assay; the luminometric methylation assay (LUMA), and the DNA methylation status of LINE-1 and Alu 1 repetitive elements using bisulfite conversion of DNA followed by PCR amplification and pyrosequencing.	Positive association between maternal U-tAs and global DNA methylation across all newborns (^3^H-methyl incorporation assay); some analyses revealed this relationship may be sex-dependent, i.e. positive for newborn males and negative for newborn females (Alu, LINE-1 and LUMA).	Pilsner et al., 2012 [55]
Women					
Women (n = 16) from Zimapán, Mexico)	7.46-90.75 μg arsenic/g creatinine in urine	PBLs	Genome-wide analysis of >14,000 promoter regions using the methylated CpG island recovery (MIRA)-microarray assay.	Distinct promoter DNA methylation patterns associated with urinary levels of iAs, MMAs and DMAs were identified, which included genes with known links to diabetes mellitus.	Bailey et al., 2013 [47]
Women from Argentinian Andes (n = 103); newborns from Bangladesh (n = 127)	U-tAs (Argentina): median = 188 μg/L; 5-95 % = 16-620 μg/L; maternal U-tAs (Bangladesh): median = 68 μg/L; 5-95 % = 20-460 μg/L	PBLs (Argentina); cord blood lymphocytes (Bangladesh)	Analysis of DNA methylation status of 10q24 region at a single nucleotide resolution using bisulfite conversion of DNA followed by hybridization to the Illumina Infinium HumanMethylation450 BeadChip.	In Argentinian women, efficient metabolizing phenotype of *AS3MT* associated methylation status of *AS3MT* and several other surrounding genes on 10q24. Similar but weaker trends observed in Bangladeshi newborns.	Engstrom et al., 2013 [49]
Native American men and women from Arizona (n = 16), Oklahoma (n = 16), North Dakota (n = 16), South Dakota (n = 16); *in vitro* exposure to arsenite (human PBMCs)	Low exposure group: U-tAs < 7.2 μg/L (mean = 5.2 μg/L; n = 8 from each region); moderate exposure group: U-tAs >14.0 μg/L; (mean = 29.2 μg/L; n = 8 from each region); human PBMCs exposed to 5.0 μM arsenite (NaAsO_2_) for 48 h	Whole blood (Native Americans); human PBMCs (*in vitro*)	In samples from Native Americans, analysis of the DNA methylation status of 48 CpG loci within *ASM3T* promoter region performed by bisulfite conversion of DNA, PCR amplification, and DNA sequencing of cloned PCR products. The DNA methylation status of *ASM3T* from PBMCs was performed using the MethyLight assay and global DNA methylation levels were determined using a 5′-mC enzyme-linked immunosorbent assay (ELISA).	A region of 30 CpG loci identified within *ASM3T* promoter that are hypomethylated in moderate exposure group vs. low exposure group. *In vitro* analyses revealed that exposure to arsenite was associated with increased *ASM3T* expression and reduced *ASM3T* promoter DNA methylation levels. Knockdown of ASM3T *in vitro* resulted in inhibition of arsenite-induced global DNA hypomethylation.	Gribble et al., 2013 [51]

HPLC-MS/MS: high-performance liquid chromatography tandem mass spectrometry

PBLs: peripheral blood lymphocytes

PBMCs: peripheral blood mononuclear cells

PCR: polymerase chain reaction

ND: non-detectable

U-tAs: total urinary arsenic (sum of iAs, MMAs, DMAs)

Our laboratory was interested in identifying genome-wide gene-specific DNA methylation changes associated with chronic iAs exposure in humans. An examination of DNA methylation patterns in peripheral blood leukocytes (PBLs) from an exposed population in Zimapán, Mexico, identified a set of 183 genes with differentially methylated promoters in women with iAs-associated skin lesions vs. women with unlesioned skin [46•]. Most of these genes had promoters that were hypermethylated in women with skin lesions (99 %), and several genes had been associated with iAs-induced diseases such as diabetes mellitus, cardiovascular disease, and cancer. The cancer-associated genes included a “tumor suppressorome” complex containing 17 genes known to be silenced in various human cancers. When these same blood samples were further analyzed in relationship to urinary measurements of iAs, MMAs, and DMAs, distinct patterns of DNA methylation associated with levels of each of these arsenicals were observed [47]. Interestingly, measurements of the percentage of glycosylated hemoglobin in the blood (%HbA1c) of these women indicated that 94 % were either pre-diabetic or diabetic, and we observed an enrichment of genes with associations with type 1 and type 2 diabetes mellitus associated with one or more of these urinary metabolites. These findings support the role of the different iAs metabolites as factors influencing the epigenome and highlight the need to investigate interindividual differences in iAs metabolism on epigenetic effects. An interesting observation is that many of the identified genes with iAs-associated DNA methylation changes have similar functionality in the cell, and thus likely possess common response elements for transcription factor binding. To explain this phenomenon, the laboratory’s current hypothesis is that toxicant-induced transcription factor occupancy influences access of DNA methyltransferases to particular regions of the genome, resulting in “DNA methylation footprints” [48].

Other studies in exposed populations have linked iAs metabolism to differences in DNA methylation patterns, including two studies that have examined the relationship between the DNA methylation patterns and polymorphisms in the arsenic (+3 oxidation state) methyltransferase (*AS3MT*) gene, which is the gene encoding the enzyme required for iAs biotransformation (Fig. 1). In chronically-exposed women living in the Argentinian Andes, a major *AS3MT* haplotype associated with more efficient iAs metabolism was associated with increased methylation of *AS3MT* and differential methylation of several other genes located on chromosome band 10q24 along with *AS3MT* [49]. Similar but weaker trends were also identified in the cord blood of Bangladeshi newborns exposed to arsenic *in utero* [49].

A separate study examined the relationship between the *AS3MT* haplotype, concentrations of urinary arsenicals, and DNA methylation levels of the tumor suppressor gene *p16*, the DNA repair gene mutL homolog 1, colon cancer, nonpolyposis type 2 (*E. coli*) (*MLH1*), and the Long Interspersed Nuclear Element 1 (LINE-1) in the blood of chronically exposed women from the Argentinian Andes [50•]. A significant positive relationship was observed between the slow-metabolizing *AS3MT* haplotype and *p16* methylation. The concentration of total urinary arsenic (U-tAs; sum of iAs, MMAs, DMAs) was associated with *p16* and *MLH1* methylation, urinary %iAs was positively associated with *p16* methylation, and urinary %DMAs was negatively associated with *p16* methylation. Interestingly, although increased urinary ratios of MMAs and/or high ratios of MMAs/DMAs are associated with increased risk for a variety of iAs-associated diseases [6], this study suggests iAs may have more toxic effects than its metabolites on the methylation status of the *p16* promoter, at least in DNA isolated from blood. No significant relationship was observed between LINE-1, used as an indicator of global DNA methylation status, and urinary arsenical measurements [50•].

One study investigated the promoter DNA methylation level of the *AS3MT* promoter in Native Americans with low vs. moderate U-tAs [51]. Out of 48 CpGs assayed in the *AS3MT* promoter, a region was identified that contained 30 hypomethylated CpGs in the moderate U-tAs group compared to the low U-tAs group, but there were no associations observed between *AS3MT* promoter methylation levels and %MMAs or %DMAs in urine. This study also revealed that short-term exposure of peripheral blood mononuclear cells (PBMCs) to sodium arsenite *in vitro* induced *AS3MT* expression, which was accompanied by a decrease in DNA methylation of the *AS3MT* promoter. In addition, *AS3MT* knockdown inhibited arsenite-induced global DNA hypomethylation in PBMCs *in vitro.* While this study established a relationship between *AS3MT* promoter methylation status and arsenic exposure in humans, further studies will be necessary to determine if these DNA methylation alterations are associated with changes in *AS3MT* expression in human populations and how these changes impact iAs biotransformation and risk of iAs-associated disease development.

Several studies have examined the DNA methylation status of targeted genes including tumor suppressors and/or global methylation of LINE-1 in the cord blood of exposed newborns. Analyses of cord blood from a cohort of newborns in Thailand exposed to varying levels of iAs *in utero* identified a positive relationship between arsenic exposure and *TP53* promoter methylation status but not LINE-1 methylation status [52]. The relationship between DNA methylation patterns of LINE-1 and *TP53* and *p16* promoters were examined in maternal and umbilical cord leukocytes isolated from iAs-exposed mother-child pairs in Bangladesh [53]. Similar trends were observed between DNA methylation trends collected from maternal and newborn blood, in which there was a positive association between maternal U-tAs levels and LINE-1 methylation and some CpG sites within the *p16* promoter. The data revealed evidence for a nonlinear dose-response relationship, as these effects were most evident in the middle tertiles of exposure. These data suggest that moderate levels of iAs, which are likely relevant to many areas endemic for arsenic poisoning, may have significant effects on DNA methylation patterning that could result in genomic alterations.

Supporting the impact of moderate levels of iAs exposure on the epigenome are changes in DNA methylation observed in a population exposed to relatively low levels of iAs. A genome-wide examination of cord blood isolated from newborns from New Hampshire revealed that while no global effects on DNA methylation were observed in relationship to maternal U-tAs levels, most of the U-tAs-associated effects were observed in CpG islands [54•]. Of the CpG islands tested, 18 % were differentially methylated among newborns associated with different maternal U-tAs levels, and the DNA methylation status of several of these CpG islands had linear relationships with maternal U-tAs [54•]. Interestingly, a potential sex-dependent relationship was observed between maternal U-tAs levels and global cord blood DNA methylation in Bangladeshi newborns [55•]. While there was a positive relationship between maternal U-tAs levels and global cord blood DNA methylation in all newborns in the study, when the newborns were divided by sex, this relationship was positive for males and negative for females. If the identified changes in DNA methylation levels are, indeed, functional and impact gene/protein expression, these alterations may influence differential susceptibility to iAs-induced disease in males and females, a feature observed in various cohorts exposed to iAs. Taken together, these studies are informative, as they reveal that even low to moderate levels of iAs exposure can impact the DNA methylation landscape. They also highlight the intriguing finding that some of these effects may be sex-dependent and suggest an association between iAs metabolism and DNA methylation patterns.

There are a few caveats to consider when interpreting the existing body of literature related to arsenical-associated changes in DNA methylation patterning. For example, although PBL DNA methylation profiles may serve as useful biomarkers for iAs exposure, disease state, and/or disease susceptibility, it is currently unknown whether PBL DNA methylation profiles reflect those that would be present in target cells of iAs toxicity such as liver, lung, and bladder. In addition, it is important to note that multiple white blood cell types are present in PBL preparations, and these cell populations may shift upon exposure to iAs or due to other environmental factors. It is also unknown if the gene-specific DNA methylation changes correspond to changes in mRNA or protein levels of their genes. Additionally, the relationship between DNA methylation and gene expression is more complex than often considered, where increased DNA methylation may be associated with increased gene expression. There is currently limited information on the stability of the DNA methylation changes over time. Also, in the case of prenatal iAs exposure, the studies presented thus far do not investigate true transgenerational effects, as iAs can readily pass through the placenta [56, 57], and thus warrant an assessment of transgenerational effects of various iAs-associated phenotypes that persist to the F3 generation and beyond. It is important to note, therefore, that while the available studies serve as a critical foundation for understanding the association between iAs exposure and the epigenome, future studies will be necessary to examine the relationship between these alterations and the development of iAs-associated disease.

## Arsenic and histone post-translational modifications (PTMs)

Histones play an essential role in the compaction and organization of the genome and in the regulation of gene activity. Histones comprise the protein component of the nucleosome, which is the fundamental repeating unit of chromatin. The nucleosome contains ~146 bp of DNA wrapped around a histone octamer comprising two copies each of the four core histones H2A, H2B, H3, and H4 [58]. Each of the four core histones contains unstructured amino-terminal (N-terminal) and carboxy-terminal (C-terminal) tails, and specific amino acids within those tails are targets of post-translational modifications (PTMs) [59]. Each histone may have multiple PTMs, which collectively comprise the “histone code” of a region and therefore influence its transcriptional competency via effects on nucleosome structure/positioning and by acting as protein-binding targets [60]. While multiple PTMs have been identified, those that are the most extensively studied and best understood in the context of transcriptional competency are histone phosphorylation, methylation, acetylation, and ubiquitination [61].

Most of the investigations into the effects of arsenicals on histone PTMs have been performed *in vitro*, in which changes in both global and gene-specific histone PTM patterns have been observed (as reviewed in [22]). Of note, several of these gene-specific histone PTM alterations have identified a relationship between arsenic exposure, the acquisition of targeted promoter histone PTM marks and DNA methylation patterns, and transcriptional competency. In at least one example, altered histone PTMs and the altered expression of a particular gene were associated with the toxic effects of arsenic. For instance, the arsenical-mediated malignant transformation of a human urothelial cell line was associated with aberrant promoter histone PTMs and overexpression of a key gene, namely wingless-type MMTV integration site family, member 5A (*WNT5A*), which is essential for the maintenance of the malignant phenotype [62].

As observed in the case of DNA methylation, recent years have witnessed an increase in the number of studies that have examined histone PTMs in iAs-exposed populations (Table 2). For instance, a positive association was observed among steel workers exposed to inhalable metal-rich particles between arsenic and nickel exposure and global PBL histone H3 lysine 4 dimethylation (H3K4me2) and histone 3 lysine 9 acetylation (H3K9ac) in PBLs [63]. A study of iAs-exposed individuals in Bangladesh revealed that some arsenic-associated changes in histone PTMs were common across all individuals, whereas others were sex-specific [64•]. For instance, U-tAs levels were positively associated with the transcription-restrictive PTM histone 3 lysine 9 dimethylation (H3K9me2) and negatively associated with the transcription-permissive histone PTM histone 3 lysine 9 acetylation (H3K9ac) in the PBLs of both men and women. The authors point out that the positive association of H3K9me2 with U-tAs is particularly interesting in light of the DNA hypermethylation of LINE-1 elements in iAs-exposed mother-child pairs in Bangladesh [53], as H3K9me2 is a marker for DNA methylation. Therefore, data from these two studies are consistent with arsenic-associated epigenetic effects that may favor overall transcriptional repression [64•]. However, in addition to trends observed for specific histone PTMs in both men and women, four other histone PTMs were identified that exhibited sex-dependent associations with levels of iAs in drinking water [64•].

**Table 2:** Recent studies examining the relationship between histone PTMs and arsenic exposure

Type of study (*in vitro*, *in vivo*, human subjects)	Arsenical exposure	Cell type analyzed	Type of histone PTM analysis and method used	Major findings	Reference
Male steel workers from Italy (n = 63)	Cumulative exposure estimated by product of arsenic content in particulate matter (PM) in air and years of employment	PBLs	Global analysis of H3K4me2 and H3K9Ac levels determined using the sandwich enzyme-linked immunosorbent assay (ELISA).	Positive association between global H3K4me2 and H3K9Ac levels with cumulative arsenic exposure.	Cantone et al., 2011 [63]
Men (n = 20) and women (n = 20) from Bangladesh	Maternal U-tAs median = 247.8 μg/g creatinine	PBMCs	Global levels of various histone PTMs determined using the sandwich enzyme-linked immunosorbent assay (ELISA).	Positive associations between U-tAs and global H3K9me2 levels and negative associations between U-tAs and global H3K9ac levels across all individuals. Additional sex-specific associations between particular histone PTMs and U-tAs identified.	Chervona et al., 2012 [64•]
*In vivo* (mice) exposure to arsenite	50 ppm NaAsO_2_ in drinking water for six months	Whole liver of male C57Bl/6 J mice	Analysis of various histone PTMs within the promoter region of *p16* gene using the chromatin immunoprecipitation (ChIP) assay. For *p16* promoter, determined DNA methylation using bisulfite conversion of DNA, followed by PCR amplification and sequencing of cloned PCR products.	Downregulation of *p16* mRNA, accompanied by increase in the transcription-restrictive modification H3K9me2 in *p16* promoter but no change in *p16* promoter DNA methylation status.	Suzuki et al., 2013 [65•]

HPLC-MS/MS: high-performance liquid chromatography tandem mass spectrometry

PBLs: peripheral blood lymphocytes

PBMCs: peripheral blood mononuclear cells

PCR: polymerase chain reaction

ND: non-detectable

U-tAs: total urinary arsenic (sum of iAs, MMAs, DMAs)

One study in mice indicated that changes in promoter PTMs are not always associated with changes in DNA methylation patterns. Long-term (6 months) exposure to iAs resulted in the downregulation of *p16* mRNA in the livers of C57BL/6 mice, which was accompanied by an increase of the transcription-restrictive PTM H3K9me2 in the *p16* promoter region but no change in promoter DNA methylation status [65•].

Together, these recent studies demonstrated that exposure to iAs is associated with genome-wide changes in histone PTMs in animals and humans and that some of these effects in humans may be sex-specific. Importantly, mechanistic *in vitro* and *in vivo* studies have provided important groundwork for studying the relationship between iAs exposure, changes in histone PTMs, and potentially toxic effects, such as the transcriptional modulation of genes implicated in disease development (e.g., tumor suppressor *p16*). The relationship between these events and iAs-associated disease development must be addressed in future work. In addition, the studies highlighted here underscore the need to examine the interplay between multiple epigenetic components that likely work in unison to mediate the toxic effects of arsenicals.

## Arsenic and microRNAs (miRNAs)

miRNAs are non-coding RNAs 21–24 nucleotides in length that are best characterized as regulators of gene activity at the post-transcriptional level, although they have been shown to regulate gene expression at the transcriptional level as well [66, 67]. At the post-transcriptional level, miRNAs bind to the 3’ untranslated region (3’ UTR) of mRNAs, which can alter mRNA function by interfering with mRNA translation into protein or by targeting mRNAs for degradation [68]. The regulation of miRNA expression is complex, as miRNA levels are controlled not only at the transcriptional level but also post-transcriptionally, where they undergo multiple processing steps to yield mature miRNAs [69]. Approximately 2000 mature miRNAs have been identified in humans [70], and by analyzing conserved miRNA binding sites within the 3’ UTR, it is believed that >60 % of protein-encoding human genes are targets of miRNAs [71].

The effects of iAs on miRNA levels have been relatively unexplored in comparison to other epigenetic modifications, particularly as they relate to chronic disease development in humans (as reviewed in [22]). Previous reviews have highlighted that most studies of the effects of arsenical exposure on miRNAs have been performed *in vitro*, and few have investigated the functional consequences of these perturbations [22, 23]. In recent years, *in vitro* studies have continued to predominate, but many are mechanistic in nature, in which miRNAs have been implicated in mediating various toxic effects of arsenicals (Table 3).

**Table 3:** Recent studies examining the relationship between miRNAs and arsenic exposure

Type of study (*in vitro*, *in vivo*, human subjects)	Arsenical exposure	Cell type analyzed	Type of miRNA analysis and method used	Major findings	Reference
*In vitro* exposure to arsenite (immortalized human bronchial epithelial cell line)	0, 10, 15, 20 μM arsenic chloride for 24 h	Human immortalized bronchial epithelial cell line BEAS-2B	Total RNA analyzed for levels of 95 cancer-related miRNAs using a miRNA qPCR array. Subsequent analysis of miR-190 levels using real-time qPCR.	Arsenite exposure correlated with dose-dependent increase in miR-190. miR-190 overexpression alone sufficient for acquisition of malignant characteristics in BEAS-2B.	Beezold et al., 2011 [77]
*In vitro* exposure to ATO (human hepatocellular carcinoma cell line)	4 μM ATO (As_2_O_3_) for 24 h	Human hepatocellular carcinoma cell line Hep-G2	Total RNA analyzed for levels of 677 human miRNAs using a miRNA PCR array. Levels of selected miRNAs analyzed using real-time qPCR.	Identified miR-29a as a likely critical mediator of ATO-mediated apoptosis and inhibition of cell growth.	Meng et al., 2011 [73]
*In vitro* exposure to arsenite (immortalized human bronchial epithelial cell line)	2.5 μM arsenite (NaAsO_2_) for 16 weeks, resulting in malignant transformation	Immortalized human bronchial epithelial cell line p53^low^ HBEC, which contains stable shRNA knockdown of TP53	Total RNA analyzed for levels of 856 human miRNAs using a miRNA PCR array. Levels of selected miRNAs also analyzed using real-time qPCR.	Malignant transformation associated with reduction of miR-200 family members; downregulation of miR-200b shown to be essential for malignant transformation.	Wang et al., 2011 [76]
*In vivo* exposure to arsenite (chick embryos); *in vitro* exposure to arsenite (human umbilical cord vein cell line)	100 nM arsenite (NaAsO_2_) injected in yolk sac of chick embryos at HH stages 6, 9, and 12; embryos harvested at HH stage 18. EA.hy926 cells exposed to 100 nM arsenite (NaAsO_2_) for 72 h	White Leghorn chick (Bovan strain) whole embryos; human umbilical cord vein cell line EA.hy926	Total RNA analyzed for miRNA levels using several methods including miRNA PCR arrays and real-time qPCR and Northern blots of selected targets.	miR-9 and miR-181b implicated as potential mediators of the toxic developmental effects of arsenic, specifically abnormal angiogenesis.	Cui et al., 2012 [75]
*In vitro* exposure to ATO (APL cell line)	Therapeutic dose of 2 μM ATO (As_2_O_3_) for 24 h	human APL cell line NB4	Total RNA analyzed for levels of 88 cancer-associated human miRNAs using a miRNA PCR array.	Exposure modulated 88 cancer-associated miRNAs, including those predicted to bind mRNAs of genes involved in cell cycle and apoptosis.	Ghaffari et al., 2012 [72]
*In vitro* exposure to arsenite (immortalized human embryonic lung fibroblasts)	1.0 μM arsenite (NaAsO_2_) for ~15 weeks, resulting in acquisition of malignant characteristics	Immortalized human embryonic lung fibroblast cell line HELF	Total RNA analyzed for miR-21 levels using real-time qPCR.	Identified ROS-sensitive pathways as inducers of miR-21. MiR-21 upregulation implicated as important event in the acquisition of malignant characteristics.	Ling et al., 2012 [78•]

APL: acute myelocytic leukemia

ATO: arsenic trioxide

HH: Hamburger-Hamilton

qPCR: quantitative polymerase chain reaction

ROS: reactive oxygen species

Some of these studies have focused on miRNAs as mediators of the apoptotic effects of arsenic trioxide (ATO) [72, 73], a chemotherapeutic agent used in the treatment of relapsed or refractory acute promyelocytic leukemia [74]. Other studies have indicated miRNAs may play roles in the diverse toxic effects of iAs. For instance, miR-9 and miR-181b were implicated as promoting abnormal angiogenesis in iAs-exposed chick embryos [75], and specific miRNAs have been implicated in iAs-mediated carcinogenesis. As an example, the reduction of miR-200b was shown to be essential for the iAs-mediated malignant transformation of TP53-deficient human bronchial epithelial cells [76]. Interestingly, the iAs-mediated miR-200b reduction was accompanied by increased methylation of the miR-200 promoter [76], highlighting the potential interplay between multiple forms of epigenetic regulation on gene expression. IAs was also found to induce miR-190 in a dose-dependent manner in a human bronchial cell line, in which miR-190 overexpression was shown to enhance malignant characteristics [77]. This study also identified a target of miR-190, namely mRNA of the PH domain leucine-rich repeat protein phosphatase (*PHLPP*) gene, which has known tumor suppressor functions. Another study identified the upregulation of miR-21 as an important event in the iAs-mediated transformation of human embryo lung fibroblast cells, which was dependent on the activation of ROS-sensitive pathways involving extracellular signal-regulated kinase mitogen-activated protein kinase (ERK/MAPK) and transcription factor nuclear factor kappa-light-chain-enhancer of activated B cells (NF-κB) [78•].

Together, these studies indicate that miRNAs may be involved in mediating multiple toxic effects of arsenic species, including the modulation of pathways that control cell growth, differentiation, and apoptosis. The studies also provide insight regarding the involvement of epigenetic modifications and cellular signaling pathways in the modulation of miRNA levels in response to iAs exposure. Future research could elucidate the mechanisms by which iAs can perturb miRNA expression levels and assess whether such expression is altered in biological samples from humans. As with the other forms of epigenetic modifications, it will be critical to confirm the functional consequences of changes in the expression of miRNAs on the expression of their transcriptional targets and to determine whether these changes ultimately influence protein abundance and function.

## Conclusions and future directions

In summary, current literature clearly indicates that arsenic exposure is associated with changes that include altered patterns of DNA methylation (both global and gene-specific), modifications of histone PTMs, and altered expression levels of miRNAs. Importantly, answers to some key questions remain unknown: (1) *What is the relationship of the epigenetic changes to disease*?-The significance of these arsenic-associated epigenetic effects on human disease development is likely dependent on a number of factors, including genotype, combined exposures to other contaminants, and other environmental influences such as nutrition; (2) *How do the epigenetic changes influence disease directly*?-While many associations have been established, it is largely unknown if there is a causal relationship between the epigenetic changes and arsenic-associated disease development, as well as the mechanisms by which these changes cause disease; (3) *How stable are the epigenetic changes and are they functional*?-It is important to consider that some epigenetic changes may be adaptive or stochastic responses that do not promote disease development. Likewise, global and/or gene-specific changes in DNA methylation patterning and histone PTMs may not be reflective of functional changes that are manifested at the protein level. Of note, it is important to consider that several major research trends have emerged in recent years. For instance, the DNA methylation studies were almost exclusively conducted on samples from human populations, whereas the miRNA studies were dominated by *in vitro* studies. These trends reveal gaps in research that must be closed in order to fully understand the molecular mechanisms that drive arsenical-associated epigenetic alterations, the consistency of these alterations across experimental platforms and study type (e.g., *in vitro* studies, *in vivo* studies, human studies), and ultimately, the relevance of these alterations to altered disease risk in exposed populations. Lastly, it will be increasingly important to examine the effects of arsenicals at a systems-wide level, where the impacts of both epigenetic and non-epigenetic alterations are examined. It is likely that the true biological picture of the epigenetic contributions to the mechanism of iAs-induced disease represents the complex interplay of a multitude of signaling events within the cell.
